# Rifaximin-Mediated Changes to the Epithelial Cell Proteome: 2-D Gel Analysis

**DOI:** 10.1371/journal.pone.0068550

**Published:** 2013-07-26

**Authors:** Caroline Schrodt, Erin E. McHugh, Mary Ann Gawinowicz, Herbert L. DuPont, Eric L. Brown

**Affiliations:** 1 Center for Infectious Diseases, the University of Texas School of Public Health, Houston, Texas, United States of America; 2 Protein Core Facility, Columbia University College, New York, New York, United States of America; 3 Internal Medicine Services, St. Luke’s Episcopal Hospital and Department of Medicine, Infectious Diseases Section, Baylor College of Medicine, Houston, Texas, United States of America; University of Cambridge, United Kingdom

## Abstract

Rifaximin is a semi-synthetic rifamycin derivative that is used to treat different conditions including bacterial diarrhea and hepatic encephalopathy. Rifaximin is of particular interest because it is poorly adsorbed in the intestines and has minimal effect on colonic microflora. We previously demonstrated that rifaximin affected epithelial cell physiology by altering infectivity by enteric pathogens and baseline inflammation suggesting that rifaximin conferred cytoprotection against colonization and infection. Effects of rifaximin on epithelial cells were further examined by comparing the protein expression profile of cells pretreated with rifaximin, rifampin (control antibiotic), or media (untreated). Two-dimensional (2-D) gel electrophoresis identified 36 protein spots that were up- or down-regulated by over 1.7-fold in rifaximin treated cells compared to controls. 15 of these spots were down-regulated, including annexin A5, intestinal-type alkaline phosphatase, histone H4, and histone-binding protein RbbP4. 21 spots were up-regulated, including heat shock protein (HSP) 90α and fascin. Many of the identified proteins are associated with cell structure and cytoskeleton, transcription and translation, and cellular metabolism. These data suggested that in addition to its antimicrobial properties, rifaximin may alter host cell physiology that provides cytoprotective effects against bacterial pathogens.

## Introduction

Rifaximin is a semi-synthetic, poorly-absorbed (<0.4%) rifamycin derivative used to treat travelers’ diarrhea due to bacterial enteropathogens and hepatic encephalopathy (HE) [1], [2], [3], [4], [5], [6]. Rifaximin also has been used for treatment of irritable bowel syndrome, small bowel bacterial overgrowth, pouchitis, and fulminant ulcerative colitis [2], [7], [8], [9], [10], [11]. The mechanisms by which rifaximin confers its broad range of intestinal effects remain largely unknown [12].

Rifaximin was first approved for use in Italy in 1987. In contrast to conventional antibiotics that have broad antimicrobial effects dramatically altering body flora rifaximin acts primarily in the gut [13]. We previously demonstrated that pretreatment of the HEp-2 epithelial cell line with rifaximin significantly reduced the ability of enteroaggregative *Escherichia coli* (EAEC) to adhere to either the epithelial cells or glass slides compared to untreated cells or cells treated with the related antibiotic rifampin [14]. In addition, epithelial cells pretreated with rifaximin had fewer detectable pro-inflammatory cytokines present in the supernatant compared to untreated controls. Finally, rifaximin pretreatment of HEp-2 cells reduced *Bacillus anthracis* internalization into lung epithelial cells (A549) but had no effect on *Shigella sonnei* internalization into cervical epithelial (HeLa) cells. These data suggested that rifaximin induced changes to supernatant cytokine profiles and interfered with the cellular process utilized by some pathogens (EAEC and *B. anthracis*) but not others (*S. sonnei*) that use a type III secretory system to gain access to the intracellular compartment [14].

By analyzing and comparing epithelial cell protein expression profiles following treatment with or without rifaximin (or with control antibiotics) we were able to identify changes in the protein expression profiles between epithelial cells in the different treatment groups as a means of understanding how rifaximin treatment altered HEp-2 physiology. Using 2-dimentional (2-D) gel electrophoresis we compared the protein expression profiles of rifaximin treated or untreated cells.

## Materials and Methods

### Cell Culture

HEp-2 cells (larynx-derived cell line obtained from the American Type Culture collection [14]) were cultured in DMEM 10% fetal bovine serum media and grown to confluency as described [14] then pretreated with rifaximin (RX), acetone (diluent control), or rifamycin (MY) (antibiotic control), or left untreated for 48 h. The media was removed, the cells washed 3X with phosphate buffered saline (PBS, pH 7.4), and the cells removed using a cell scraper. One Complete™ Mini Protease inhibitor cocktail tablet (Roche Diagnostics, Indianapolis, IN) dissolved in 500 µl was added to the cells and respective pellets were then frozen at −80°C and shipped on dry ice to Kendrick Labs, Inc. (Madison, WI) for analysis by 2-dimensional (2-D) gel electrophoresis using the carrier ampholine method of isoelectric focusing.

### 2-D Gel Electrophoresis

Isoelectric focusing was carried out in a glass tube with an inner diameter 3.3 mm using 2.0% pH 3.5–10 mix 4 L ampholines for 20,000 volt-hours. One hundred ng of an IEF internal standard, tropomyosin, was added to each sample. This protein migrated as a doublet with a lower polypeptide spot of 33,0000 Da and pI 5.2. A surface pH electrode was used to determine the gel pH gradient plot for this set of ampholines. After equilibration for 10 min in Buffer ‘O’ (10% glycerol, 50 mM dithiothreitol, 2.3% SDS, and 0.0625 M tris, pH 6.8) each tube gel was sealed to the top of a stacking gel and overlaid with a 10% acrylamide slab gel (1.00 mm thick). SDS slab gel electrophoresis was then carried out for 5 h at 25 mA/gel. The following proteins were used as molecular weight standards: myosin (220,000), phosphorylase A (94,000), catalase (60,000), actin (43,000), carbonic anhydrase (29,000), and lysozyme (14,000). These standards appeared along the basic edge of the silver stained 10% acrylamide slab gels. The silver-stained gels were then dried between sheets of cellophane with the acid edge to the left.

### 2-D Gel Image Analysis

To conduct comparisons between treatment groups, duplicate gels obtained from each sample were scanned with a laser densitometer. The scanner was checked for linearity prior to scanning with a calibrated Neutral Density Filter Set. The images were analyzed using Progenesis SameSpots software (version 4.0, Nonlinear Dynamics, Durham, NC) and Progenesis PG240 software (version 2006, Nonlinear Dynamics, Durham, NC). The general method of computerized analysis for these pairs included image warping followed by spot finding, background subtraction (average on boundary), matching, and quantification in conjunction with detailed manual checking. Spot percentages would be equal to spot integrated density above background (volume) expressed as a percentage of total density above background of all spots measured. Differences were defined as fold-change of spot percentages.

### Protein Digestion and Identification

Proteins up- or down-regulated by more than 1.7-fold were cut out, washed, digested with trypsin, and analyzed by MALDI-MS at the Protein Chemistry Core Facility at Colombia University. Gel spots were transferred to clean tubes, hydrated with water, and plastic coating was removed with clean forceps. Spots were prepared for digestion by washing twice with 100 µL of 0.05 M Tris/30% acetonitrile (pH 8.5) for 20 min. After washing, gel spots were dried for 30 minutes in a Speed-Vac concentrator.

Gels were digested in 0.08 µg modified trypsin (sequencing grade, Roche Molecular Biochemicals) in 13–15 µL of 0.025 M Tris (pH 8.5) and placed on a heating block at 32°C overnight. Peptides were extracted with 2×50 µL 50% acetonitrile/2% TFA; the combined extracts were dried and suspended in matrix solution (10 mg/mL solution of 4-hydroxy-α-cyanocinnamic acid in 50% acetonitrile/0.1% TFA, internal standards: angiostensin and ACTH peptide).

Resuspended digests were spotted on sample plates and allowed to dry. MALDI mass spectrometric analysis was done on the digests using an Applied Biosystems Voyager DE Pro mass spectrometer. Peptide masses were subsequently entered into search programs utilizing the NCBI and/or GenPept databases for protein match. Error tolerance was set at 0.5 Da for average masses.

## Results

**Figure 1:**
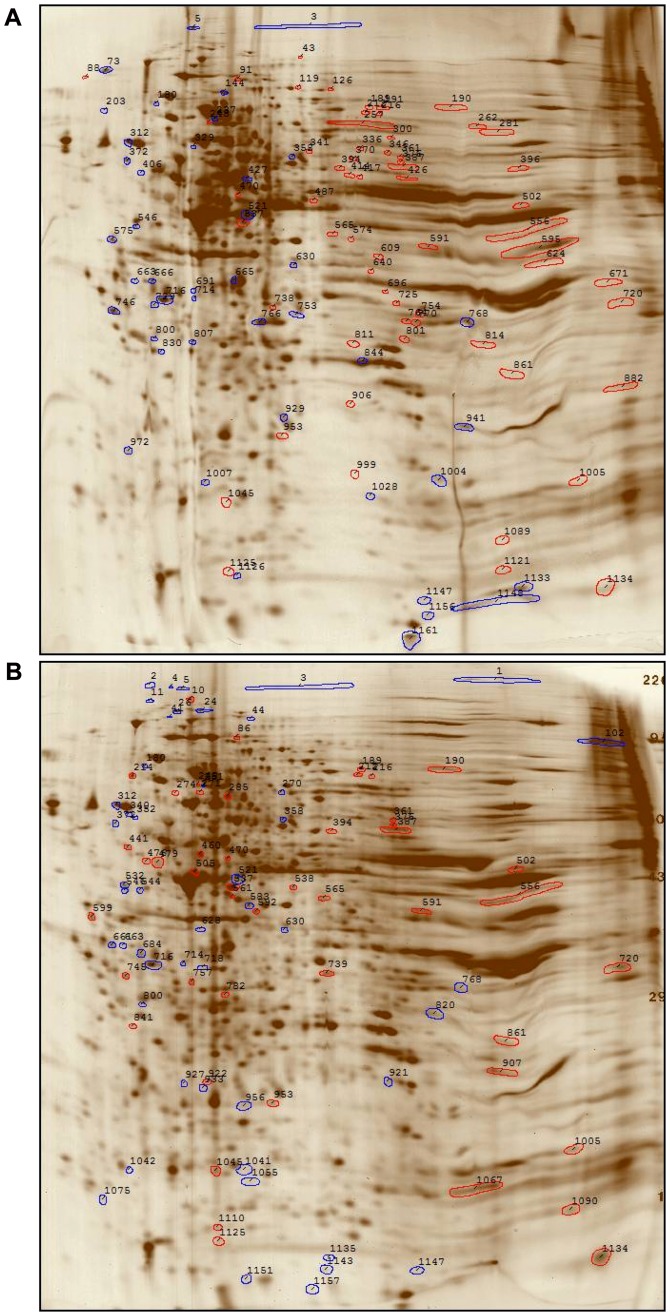
2-D gel analysis. (A) HEp-2 cell 2-D profile of rifaximin (RX)-treated vs. untreated cells. Spots decreased in RX vs. untreated cells are outlined in blue and up-regulated spots in RX vs. untreated are outlined in red. See Table 1 for spot measurements. (B) HEp-2 cell 2-D profile of RX-treated vs. rifamycin (MY)-treated cells. Spots decreased in RX vs. MY-treated cells are outlined in blue and up-regulated spots in RX-treated vs. MY-treated are outlined in red. See Table 1 for spot measurements.

The HEp-2 cell proteome was analyzed following cell treatment with either rifaximin, acetone (control), rifamycin (control antibiotic), or left untreated. A total of 1,164 spots were analyzed using the Progenesis SameSpots software and the Progenesis PG240 software. Representative gels analyzed for differential expression between cells treated with rifaximin compared to rifamycin or rifaximin compared to media alone are shown (Figure 1A and 1B, respectively). Acetone treated cells yielded a profile similar to that of rifamycin-treated cells or untreated cells (data not shown). Comparison of the 2-D gel profile of HEp-2 cells treated with rifaximin relative to the profiles defined for HEp-2 cells in each of the respective control groups identified 184 polypeptide spots differentially up- or down-regulated, however, only 36 spots were selected for sequencing based on their differential expression levels. Of the 36 protein spots, 26 spots sequenced were up- or down-regulated in rifaximin-treated cells by ≥2.0-fold relative to the expression profile in both control groups. Eight protein spots were up- or down-regulated by ≥2.0 in one of the two control groups analyzed. Spot 180 (intestinal-type alkaline phosphatase) was up-regulated and spot 591 (protein haymaker) was down-regulated, relative to both control groups by ≥1.7 (Table 1). A total of 15 protein spots were down-regulated and 21 protein spots were up-regulated, in the rifaximin treated group compared to cells treated with rifamycin, media alone, or acetone (data not shown) (Table 1). Spots 406 (NDRG1) and 487 (no match) (Table 1) were not significantly different in either untreated cells or rifamycin-treated cells (relative to the expression profile observed for rifaximin-treated cells) and are therefore not discussed further. Spots corresponding to proteins significantly up- or down-regulated were cut out, digested with trypsin, and analyzed by MALDI-MS at the Protein Chemistry Core Facility at Colombia University (Tables 2 and 3) and classified by their respective functions (Table 4). Of the spots analyzed, 26 unique polypeptides were positively identified, two polypeptides were tentatively identified as tubulin beta chain and heat shock protein HSP 90, and 11 polypeptides were unidentified. Most proteins positively identified were associated with either cell transcription/translation (n = 11), cell structure (n = 3), or metabolism (n = 3) (Table 4).

**Table 1:** 2-D gel analysis profile comparing protein expression profiles between rifaximin (RX), rifamycin (MY), and untreated HEp-2 cells.

Spot #	pI	MW	AverageRX Spot %	Average AcetoneSpot %	AverageMY Spot %	Average MediaSpot %	RX vs MYDifference	RX vs MYT-test (p)	RX vs MediaDifference	RX vs MediaT-test (p)
358	6.5	58,396	0.008	0.013	0.013	0.015	1.7	0.014	2.0	0.006
406	5.3	54,020	0.004	0.012	0.010	0.008	2.2	0.033	1.7	0.436
546	5.3	40,646	0.009	0.015	0.018	0.017	2.1	0.023	2.0	0.008
716	5.5	31,437	0.192	0.376	0.351	0.419	1.8	0.024	2.2	0.039
190	7.6	80,609	0.111	0.062	0.016	0.049	*−6.9*	0.010	*−2.3*	0.045
470	6.1	46,443	0.007	0.001	0.002	0.001	*−3.5*	0.055	*−6.1*	0.031
487	6.7	44,706	0.013	0.004	0.003	0.008	*−4.4*	0.109	*−*1.6	0.292
1045	6.0	16,366	0.060	0.006	0.006	0.018	*−9.5*	0.032	*−3.4*	0.061
1125	6.0	12,650	0.027	0.003	0.005	0.008	*−5.1*	0.218	*−3.5*	0.270
3	6.6	191,601	0.109	0.120	0.187	0.247	1.7	0.012	2.3	0.007
5	5.7	188,002	0.022	0.038	0.081	0.073	3.7	0.028	3.3	0.046
180	5.4	82312	0.007	0.008	0.013	0.013	1.8	0.008	1.8	0.005
312	5.2	65,196	0.087	0.110	0.144	0.176	1.7	0.007	2.0	0.000
372	5.2	57,825	0.029	0.060	0.070	0.098	2.4	0.036	3.4	0.025
630	6.5	35,676	0.007	0.006	0.022	0.014	3.3	0.127	2.2	0.043
663	5.3	33,832	0.005	0.010	0.016	0.018	3.3	0.039	3.8	0.056
714	5.7	31,602	0.005	0.003	0.009	0.012	1.7	0.001	2.3	0.000
768	7.7	28,738	0.016	0.012	0.117	0.048	7.2	0.011	2.9	0.023
800	5.4	27,641	0.003	0.005	0.011	0.012	3.3	0.030	3.7	0.287
1147	7.5	11,431	0.008	0.009	0.029	0.020	3.4	0.172	2.4	0.003
189	7.1	80,609	0.013	0.014	0.004	0.005	*−3.6*	0.181	*−2.4*	0.022
212	7.1	78,345	0.017	0.027	0.001	0.005	*−20.1*	0.018	*−3.4*	0.057
216	7.2	77,778	0.005	0.006	0.001	0.001	*−8.2*	0.009	*−5.6*	0.013
248	5.8	74,049	0.005	0.002	0.002	0.002	*−2.4*	0.001	*−2.6*	0.005
361	7.3	60,340	0.008	0.009	0.001	0.003	*−5.9*	0.012	*−2.4*	0.024
376	7.3	57,463	0.010	0.008	0.002	0.002	*−5.1*	0.063	*−5.9*	0.136
394	6.9	54,820	0.022	0.015	0.011	0.006	*−2.0*	0.042	*−3.7*	0.032
502	8.1	43,138	0.035	0.070	0.008	0.011	*−4.3*	0.286	*−3.1*	0.339
556	8.1	39,866	0.390	0.330	0.141	0.224	*−2.8*	0.016	*−1.7*	0.049
565	6.8	39,529	0.021	0.027	0.008	0.006	*−2.8*	0.003	*−3.8*	0.001
591	7.5	37,868	0.039	0.041	0.022	0.021	*−1.8*	0.046	*−1.9*	0.042
720	8.7	31,036	0.135	0.106	0.023	0.044	*−5.9*	0.002	*−3.1*	0.003
861	8.0	25,095	0.101	0.100	0.022	0.026	*−4.6*	0.201	*−3.9*	0.199
953	6.4	21,058	0.063	0.039	0.021	0.011	*−3.0*	0.137	*−5.8*	0.089
1005	8.4	17,656	0.128	0.152	0.021	0.039	*−6.1*	0.124	*−3.3*	0.278
1134	8.6	11,817	0.387	0.411	0.107	0.016	*−3.6*	0.066	*−24.5*	0.037

Reference spot numbering, pI, and MW are provided for polypeptide spots analyzed in samples RX, MY, and Media. The differences are calculated from spot percentages (individual spot density divided by total density of all measured spots). Polypeptide spots increased in MY and Media vs. RX by a fold increase of ≥1.7 and p values <0.05 are shown in bold. Bold indicates down-regulation in samples treated with Rifaximin vs. MY or Media. Spots decreased in MY and Media vs. RX by a fold decrease of ≤ −1.7 and p value <0.05 are in italics. Italics indicates up-regulated spots identified in samples treated with Rifaximin vs. MY or Media. Spot percentages are given to indicate relative abundance. Note that the p values are for n = 2 gels/sample. A total of 1,164 spots were analyzed.

**Table 2:** Identification of down-regulated polypeptides.

Spot #	Protein	# of peptides used (%sequence Coverage)	MS-Fit MOWSEScore	MascotScore	ExpectedValue
358	Tubulin Beta chain (P07437)	12 (44)	2.80E+04	76	5.50E-04
	pre-mRNA processing factor 19 (Q9UMS4)	10 (42)	8.22E+04	60	2.00E-02
406	Protein NDRG1 (Q92597)	17 (50)	5.74E+08	93	1.00E-05
546	40S ribosomal protein SA (P08865)	9 (32)	2.29E+02	58	3.60E-02
716	Annexin A5 (P08758)	24 (63)	1.06E+10	166	5.10E-13
3	Carbamoyl-phosphate synthase (P31327)	19 (17)	4.63E+11	35	5.80E+00
5	Hypoxia up-regulated protein 1 (Q9Y4L1)	16 (18)	6.90E+05	84	8.80E-05
180	Intestinal-type alkaline phosphatase (P09923)	19 (46)	3.46E+08	117	4.00E-08
312	Protein disulfide isomerase (P07237)	19 (52)	5.43E+06	186	5.10E-15
372	Histone-binding protein RbbP4 (Q09028)	16 (58)	1.17E+04	186	5.10E-15
630	*Tentative:* Tubulin beta chain (P07437)	7 (26)	3.43E+02	nm	nm
663	Deoxyribonuclease-1 (bovine) (likelycontaminate from bovine serum media) (P00639)	6 (27)	1.49E+05	66	1.50E-02
714	No Match	nm	nm	nm	nm
768	Guanine nucleotide-binding proteinsubunit beta-2-like-1 (P63244)	17 (66)	6.15E+08	159	2.50E-12
800	Syntaxin-6 (O43752)	8 (52)	6.57E+11	79	2.70E-04
	No match				
1147	Histone H4 (P62805)	3 (29)	1.62E+02	32	1.30E+01
	No match				

**Table 3:** Identification of up-regulated polypeptides.

Spot #	Protein	# of peptides used (%sequence Coverage)	MS-Fit MOWSEScore	MascotScore	ExpectedValue
190	Far upstream element-binding protein 1 (Q96AE4)	17 (33)	6.15E+09	90	1.80E-05
	*Tentative:* heat shock protein HSP 90 alpha (P07900)	10 (25)	1.02E+05	nm	nm
470	No match	nm	nm	nm	nm
487	No match	nm	nm	nm	nm
1045	No match	nm	nm	nm	nm
1125	No match	nm	nm	nm	nm
189	Bifunctional 3′-phosphoadenosine5′-phosphosulfate synthase 1 (O43252)	19 (40)	8.06E+09	83	9.30E-05
212	Phenylalanyl-tRNA synthetase beta chain (Q9NSD9)	15 (33)	1.07E+07	90	1.80E-05
216	Phenylalanyl-tRNA synthetase beta chain (Q9NSD9)	13 (25)	6.03E+05	52	1.40E-01
248	No match; poor spectrum	nm	nm	nm	nm
361	Tubulin alpha 1B chain (P68363)	10 (29)	6.20E+05	69	2.70E-03
376	WD40 repeat-containing protein SMU1 (Q2TAY7)	13 (39)	3.52E+06	107	4.00E-07
	annexin A11 (P50995)	9 (21)	1.41E+04	44	8.60E-01
394	Tubulin alpha 1B chain (P68363)	14 (47)	1.44E+08	88	3.20E-05
	Fascin (Q16658)	12 (38)	3.58E+06	65	7.00E-03
502	Phosphoglycerate kinase 1 (P00558)	8 (25)	9.93E+02	46	5.60E-01
556	Heterogeneous nuclear ribonucleoproteinC1/C2 (P07910)	6 (30)	3.05E+02	55	6.60E-02
565	Poly(rc)-binding protein 2 (Q15366)	13 (60)	3.45E+06	132	1.30E-09
591	Protein Haymaker (O96008)	10 (54)	7.61E+03	89	2.40E-05
720	No match	nm	nm	nm	nm
861	3-hydroxyacyl-CoA dehydrogenase type 2 (Q99714)	7 (38)	3.79E+04	54	9.00E-02
953	40S ribosomal protein S7 (P62081)	7 (42)	2.89E+02	68	2.90E-03
1005	No match	nm	nm	nm	nm
1134	No match	nm	nm	nm	nm

**Table 4:** Identification of Proteins.

Functional Group	Protein Name (Spot Number, SwissProt Accession, Average Molecular Weight in Daltons)
Structural	Tubulin beta chain (Spot 358 and Tentative 630, P07437, 49670.82)
	Tubulin alpha 1B chain (Spot 394, P68363, 50151.63)
	Fascin (361 and 394, Q16658, 54398.81)
Transcription/Translational	Aspartyl-tRNA synthetase (394, P14868, 57136.22)
	Histone-binding protein RbAp48 (Spot 372, Q09028, 47524.51)
	Far upstream element-binding protein 1 (190, Q96AE4, 67429.19)
	Histone H4 (1147, P62805, 11236.15)
	Guanine nucleotide-binding protein subunit beta-2-like1 (768, P63244, 34945.54)
	40S ribosomal protein SA (546, P08865, 32722.88)
	40S ribosomal protein S7 (953, P62081, 22126.85)
	Heterogeneous nuclear ribonucleoprotein C1/C2 (556, P079010, 33538.81)
	3-hydroxyacyl-CoA dehydrogenase type 2 (861, Q99714, 26791.89)
	Phenylalanyl-tRNA synthetase beta chain (212 and 216, Q9NSD9, 66115.61)
	Poly(rc)-binding protein 2 (565, Q15366, 38580.07)
	WD40 repeat-containing protein SMU1 (376, Q2TAY7, 57412.70)
DNA binding	Pre-mRNA processing factor 19 (358, Q9UMS4, 55049.60)
Protein binding	Annexin A5 (716, P08758, 35805.58)
	Protein NDRG1 (406, Q92597, 42835.44)
Intracellular trafficking	Syntaxin-6 (800, O435752, 29175.95)
Cytokinesis	Annexin A11 Tentative (376, P50995, 54390)
Stress response, protein folding	Tentative Heat shock protein HSP 90-alpha (190, P07900, 84528.52)
	Hypoxia up-regulated protein 1 (5, Q9Y4L1, 107659.97)
	Protein disulfide isomerase precursor (312, P07237, 55294.02)
Metabolism	Bifunctional 3′-phosphoadenosine 5′-phosphosulfate synthase 1(189, O43252, 70833.15)
	Phosphoglycerate kinase 1 (502, P00558, 44483.49)
	Carbamoyl-phosphate synthase (3, P31327, 160549.19)
Other	Haymaker (591,O96008, 37893.10)

## Discussion

We previously demonstrated that rifaximin pre-treatment significantly altered the attachment pattern of EAEC to HEp-2 cells and diminished the internalization of *Bacilus anthracis* into A549 cells. In addition, rifaximin pretreatment diminished the number of proinflammatory cytokines detected in the supernatants of treated cells [14]. These observations suggested that rifaximin exerted protective effects beyond its antibiotic properties. As a result we further examined the effects of rifaximin on HEp-2 cells by characterizing rifaximin-mediated effects at the protein level by 2-D gel analysis of HEp-2 cells treated in the presence of rifaximin compared to profiles observed for untreated cells or cells treated with either rifamycin or acetone (rifaximin diluent). 2-D gel electrophoresis analysis demonstrated that the protein expression profile of HEp-2 epithelial cells treated with rifaximin differed compared to the expression profile observed for HEp-2 cells treated with acetone, rifamycin, or left untreated (Figure 1, Table 1). Of the protein spots analyzed by MALDI-MS, 15 proteins were down-regulated in rifaximin-treated cells by >1.7-fold compared to the expression profile in the control groups, including the up-regulation of annexin A5, intestinal-type alkaline phosphatase, histone H4, and histone-binding protein RbAp48 (Table 2), and 21 spots were up-regulated by >1.7-fold including heat shock protein HSP90α (tentative) and fascin (Table 3).

Increased annexin A5 (annexin V) expression is a marker for apoptosis [15] and annexin V was down-regulated in rifaximin pretreated cells. Brest *et al.* [16] observed that Afa/Dr expression by diffusely adhering *E. coli* (Afa/Dr DAEC) decreased polymorphonuclear leukocyte (PMN) phagocytosis levels while inducing apoptosis associated with increased annexin V expression [16]. In addition, cycle inhibiting factor (Cif)-expressing EPEC induced delayed apoptosis in intestinal epithelial (IEC-6) cells [17]. Cif is also expressed by enterohemorrhagic *E. coli* (EHEC) strains [18], [19] and Samba-Louaka *et al.* [17] demonstrated that increased annexin V expression levels were associated with apoptosis after IEC-6 cells were cultured in the presence of Cif-expressing EPEC. Furthermore, Figueiredo *et al.* [20] demonstrated that enterohemolysin (EHly) induced apoptosis of human intestinal epithelial cells (Caco-2 and HT-29) in association with increased annexin V expression and Fernandez-Prada *et al.* [21] demonstrated that alpha-hemolysin expressing EAEC and cytodetaching *E. coli* induced oncosis in human monocyte-derived macrophages and apoptosis in J774 murine macrophages. These data suggested that rifaximin-mediated reduction in annexin V expression may protect cells from bacterially-induced apoptosis.

Intestinal-type alkaline phosphatase (IAP) is an enzyme that hydrolyzes monophosphate esters and detoxifies lipopolysaccharides (LPS) and is found in areas of the small and large intestines, both inside the lumen and inside intestinal epithelial cells [22], [23]. The involvement of IAP as a mucosal defense factor in the intestines has been widely documented, however, the exact mechanism(s) of action remain undefined [23], [24], [25]. Malo *et al.* [26] demonstrated that the intestinal flora of IAP knock-out (IAP-KO) mice differed from the flora of wild-type controls (IAP-WT) and contained lower numbers of anaerobic and aerobic bacteria recoverable from stools. Furthermore, IAP-KO mice supplemented with IAP after antibiotic treatment restored healthy gut microbiota and prevented the growth of pathogenic *Salmonella typhimurium* [26]. In a separate study, Tuin *et al.* [27] demonstrated that IAP was decreased in patients with inflammatory bowel disease, a disease sometimes treated with rifaximin. Interestingly in our study, IAP expression was down-regulated in cells pretreated with rifaximin suggesting that IAP may not be involved in rifaximin-mediated cytoprotection. This may also be the case for histone H4 that was down-regulated following rifaximin treatment. Some members of this protein family possess bactericidal properties, for example, a histone H4-derived peptide (H4_86–100_) possessed Gram-negative (*E. coli*, *Pseudomonas aeruginosa*) and Gram-positive (*Staphylococcus aureus*, *Bacillus subtilis*) bactericidal properties [28] similar to other histones H1 [29], [30], H2A [31], [32], H2B [33], H3, and H4 [34]. However, rifaximin-mediated down-regulation of histone-binding protein rbbp4 (RbAp48) (a WD40 protein family member [35] with various functions, including mediating chromatin metabolism and assembly, Ras signaling, and cytoskeletal reorganization) has also been shown to bind human histone H4. This is significant since increased RbAp48 expression was associated with increased K-Ras activity resulting in cytoskeletal disruption, decreased cell size, reduced cellular protrusions, and a higher nuclear:cytoplasmic ratio [36]. Nicolas *et al.* [37] reported that RbAp48 may be associated with decreased transcriptional expression of E2-F genes during the G1 cell phase, indicating one mechanism whereby RbAp48 may indirectly modulate mammalian cell proliferation. Rifaximin-mediated reduction of RbAp48 further suggested that cytoprotection was likely due do modifications to the expression levels of proteins associated with the cytoskeleton and cellular integrity. This observation is further supported by the observation that fascin, an organizational protein that bundles actin in cells [38] and highly expressed in colorectal adenocarcinomas and in patients with inflammatory bowel disease [3], [38] was up-regulated following rifaximin treatment.

Heat shock protein HSP90α is one of the cytoprotective heat shock proteins produced in response to cellular stresses or environmental changes [39]. In this study, HSP90α was tentatively identified and up-regulated in cells pretreated with rifaximin and heat-shock proteins have also been shown to be modulators of apoptosis [40]. Specifically, HSP90α was shown to potentially induce or inhibit apoptosis via two distinct methods [40]. One mechanism involved prevention of apoptosome formation and subsequent caspase-9 formation [40] as a result of HSP90α binding to Apaf-1 [41], [42], [43]; and the second by preventing apoptosis via HSP90α interaction with NF-κB [40], RIP-1 kinase, and Akt [44], [45]. Zheng *et al.* [46] demonstrated that neutrophil apoptosis following exposure to *E. coli* strain ATCC 25922 and *S. aureus* was associated with increased expression of HSP60 and 70, but not HSP90α. Macrophage ingestion of apoptotic neutrophils increased cytokine production of TNFα and FcyRI surface expression as a result of stimulation by HSP60 and 70 [46]. Further research would need to be done to confirm the presence and identification of HSP90α and to determine if the up-regulation of HSP90α in this study contributed to the ability of rifaximin to protect cells against bacterial invasion or attachment.

There is a relatively wide variation among the functions of the identified proteins (Table 4), suggesting that rifaximin may alter cell physiology in various ways, some of which may alter the ability of EAEC to adhere to epithelial cell surfaces and other changes that may confer protection by preventing pathogen-induced cytoskeletal changes resulting in the prevention of a variety of gastrointestinal diseases. Many of the proteins identified primarily function in controlling cell structure and the cytoskeleton, transcription and translation, and cellular metabolism. It is possible that rifaximin-induced alterations to the protein expression profiles are responsible for the amelioration of some of the symptoms attributed to travelers’ diarrhea and other gastrointestinal diseases.

By characterizing the protein expression profiles of cells pretreated with rifaximin different uses for rifaximin can be developed. This knowledge will also provide insight into the mechanisms by which rifaximin may protect the gut against infectious agents and how it may prevent or diminish symptoms of disease mediated unrelated to enteric pathogens as exemplified by hepatic encephalopathy, irritable bowel syndrome and inflammatory bowel disease where this drug appears to have effects.
